# Expatriates' Cultural Intelligence Mediates the Relationship Between Lifestyle and Performance: A Cross‐Sectional Analysis and a Longitudinal Pilot Study

**DOI:** 10.1002/brb3.70576

**Published:** 2025-05-26

**Authors:** Keisuke Kokubun, Kiyotaka Nemoto, Yoshinori Yamakawa

**Affiliations:** ^1^ Graduate School of Management Kyoto University Kyoto Japan; ^2^ Department of Psychiatry, Institute of Medicine University of Tsukuba Tsukuba Japan; ^3^ Institute of Innovative Research Institute of Science Tokyo Meguro Tokyo Japan; ^4^ ImPACT Program of Council for Science, Technology and Innovation (Cabinet Office, Government of Japan) Chiyoda Tokyo Japan; ^5^ Office for Academic and Industrial Innovation Kobe University Kobe Japan; ^6^ Brain Impact Kyoto Japan

## Abstract

**Introduction:**

Cultural intelligence (CQ) has been attracting increasing attention in recent years as a necessary caability for adapting to a different culture and improving expatriate performance (EP). However, the methods for improving this intelligence have not been fully elucidated, and in particular, the relationship with lifestyle (LS) has hardly been clarified.

**Methods:**

In this paper, we therefore conducted a cross‐sectional analysis using questionnaire response data obtained from 184 Japanese expatriates working for Japanese subsidiaries overseas in Study 1, and in Study 2, we measured changes in 15 of them as an exploratory pilot study after using a smartphone app with health promotion functions for two weeks.

**Results:**

The results showed that CQ mediated the relationship between LS and EP in both Study 1 and Study 2.

**Conclusion:**

The results of this study suggest that a health science approach that improves LS can be effective in improving the EP of businesspeople working in different cultural environments through improving their CQ.

## Graphical abstract

The results of this study suggest that a health science approach that improves lifestyle (LS) can be effective in improving the expatriate performance (EP) of businesspeople working in different cultural environments through improving their cultural intelligence (CQ).

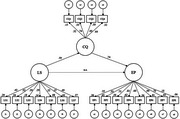

## 1 Introduction

Cultural intelligence (CQ), which is defined as an individual's ability to function and manage effectively in a culturally diverse environment (Ang et al. 2007), has attracted attention as a capability that influences the intercultural adaptation and performance of overseas workers and students, including their intention to leave, and empirical studies have been conducted (Chew et al. 2021; Jyoti and Kour 2017; Nunes et al. 2017; Ren et al. 2021; Setti et al. 2022). In addition, previous studies have shown that CQ is associated with lifestyle (LS) (Kokubun, Nemoto, et al. 2023; Kokubun, Nemoto, et al. 2025) and that LS is associated with expatriate performance (EP) (Truman et al. 2011; Fujimoto 2016; Naithani 2016; Prestes et al. 2016). However, the relationship between LS, CQ, and EP has not yet been examined. In light of the claims to understand CQ from a neuroscientific perspective (Rockstuhl et al. 2010; Ang et al. 2015), it is expected that CQ is influenced by LS and determines EP, just as the brain does (Wu et al. 2008; Gomez‐Pinilla 2011; Cho et al. 2012; Hötting et al. 2016; Mandolesi et al. 2017).

This prediction is strengthened in light of the conservation of resources (COR) theory (Hobfoll 1989). Expatriates who are forced to face various new things in a cross‐cultural environment have CQ as one of their psychological resources and use it in their daily work. If CQ is lacking, it becomes difficult to deal with things, and EP decreases. At this time, if LS exists and is recharged by being converted into CQ, the amount of resource is maintained, preventing the decline of EP. There have been recent attempts to integrate expatriate CQ into COR theory (Fu and Charoensukmongkol 2023; A. S. Y. Chen et al. 2024). COR mechanism also worked for overseas subsidiaries during COVID‐19, when they suffered from resource shortages and fears of infection (Kokubun et al. 2022; Kokubun, Ino, et al. 2025).

**FIGURE 1:**
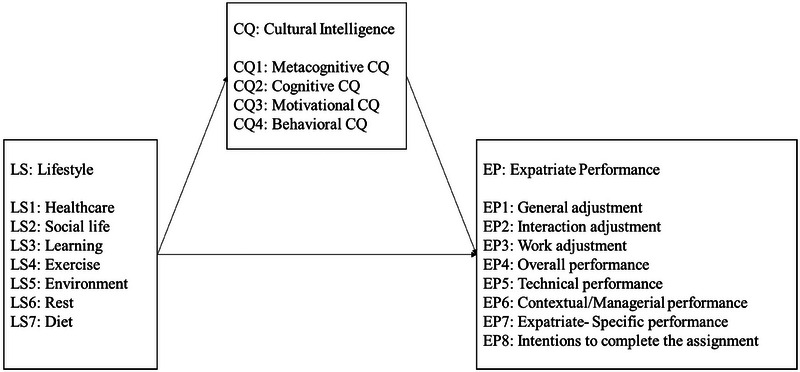
Analytical model of this study. CQ = cultural intelligence, EP = expatriate performance, LS = lifestyle.

Therefore, in this study, we clarify the relationship between LS, CQ, and EP through cross‐sectional and pilot longitudinal studies. Specifically, as shown in Figure 1, we show that CQ mediates the relationship between LS and EP. The current research bridges cross‐cultural management research and health science research, contributing to the relevant academic communities in an interdisciplinary manner.

## 2 Literature Review

Previous studies have shown that expatriates' cross‐cultural adaptation is related to performance (L. Y. Lee and Sukoco 2010; Malek and Budhwar 2013) and early termination of assignment (Caligiuri 1997, Kraimer and Wayne 2004). In addition, cross‐cultural adaptation, performance, and early termination are all major outcome variables in expatriate research, and many studies have shown their relationship with CQ (Jyoti and Kour 2017; Nunes et al. 2017; Chew et al. 2021; Setti et al. 2022). Therefore, in this study, we define cultural adjustment, performance, and intentions to complete the assignment collectively as EP. On the other hand, healthcare, social life, learning, exercise, environment, rest, and diet are included as seven superordinate concepts in the “BHQ Actions” (Brain Impact Association 2024), which are 18 guidelines for maintaining brain health formulated by researchers involved in brain research, and their relationship with CQ has also been confirmed (Kokubun, Nemoto, et al. 2023; Kokubun, Nemoto, et al. 2025). In this study, these seven concepts are collectively defined as LS.

CQ is defined as an individual's ability to function and manage effectively in a culturally diverse environment and consists of four dimensions: Metacognitive CQ, Cognitive CQ, Motivational CQ, and Behavioral CQ (Ang et al. 2007). Metacognitive CQ refers to the ability to adjust one's thoughts in a cross‐cultural environment. Therefore, individuals with high Metacognitive CQ can flexibly and highly apply cultural knowledge in diverse cultural interactions. Cognitive CQ reflects knowledge of norms, practices, and conventions used in various cultural environments. Individuals with high Cognitive CQ interact better with people from different cultures and understand similarities and differences between cultures. Motivational CQ is the ability to exert effort and energy to adapt to different cultures. High Motivational CQ leads to focused and sustained efforts to adapt and adjust to different cultural environments. Finally, Behavioral CQ refers to exhibiting appropriate behaviors in diverse cultures. High behavioral CQ includes verbal and nonverbal behaviors, such as culturally appropriate words, tone, gestures, facial expressions, and body language (Ang et al. 2007).

Previous studies have shown that CQ correlates with intercultural adaptation and performance in expatiates working for a multinational company (Jyoti and Kour 2017; Nunes et al. 2017; Chew et al. 2021; Setti et al. 2022). Recent studies have shown that CQ may enhance sales performance in cross‐cultural markets (Pandey and Charoensukmongkol 2019; Zhou and Charoensukmongkol 2022), improve the efficiency and work performance of global virtual teams (Presbitero 2021), enhance airline cabin crew's intercultural communication skills and attention to service (Suthatorn and Charoensukmongkol 2018), and enhance innovative work behaviors of multinational employees (Afsar et al. 2021). CQ is also related to a variety of individual and organizational outcomes beyond those of expatriates and multinational companies (A. P. Lee et al. 2023). For example, CQ has been shown to correlate with work engagement (Ramalu and Subramaniam 2019) and thriving and retention among international teachers (Ren et al. 2021). Furthermore, CQ has been shown to be effective in adapting to the diversity faced by people who are transferred to another state within the country (Kadam et al. 2021). Therefore, training methods to improve CQ have been proposed (Earley and Peterson 2004; Rehg et al. 2012; Philip et al. 2023). For example, the use of immersive virtual reality (VR) technology as a training device has been shown to be effective for CQ development (Philip et al. 2023). However, to the best of our knowledge, the only studies that have examined the relationship between LS and CQ are Kokubun, Nemoto, et al. 2023 and Kokubun, Nemoto, et al. 2025. They based their model on the idea that CQ is related to learning (Kokubun, Nemoto, et al. 2025) and brain conditions (Kokubun, Nemoto, et al. 2023). The composition of the four facets of CQ is very similar to the Self‐Regulated Learning (SRL) strategy theory which involves the learner's metacognitive, cognitive, motivational, and behavioral engagement (Zimmerman, 1990). Individuals who seek a certain level of health employ SRL strategies to self‐regulate their health using self‐care strategies, set reasonable health goals, and monitor feedback on the effectiveness of the strategies to achieve the goals (Clark and ​​Zimmerman, 2014). Therefore, the learning aspect of CQ may enable the selection of healthy LS and may form a feedback loop through which LS may influence CQ (Kokubun, Nemoto, et al. 2025).

Meanwhile, previous studies have also shown that the brain conditions of healthy people, as measured by gray matter volume, are associated with a balanced diet (Kokubun and Yamakawa 2019), LS (Kokubun et al. 2021), and social performance (Kokubun, Yamakawa, et al. 2023). Research has also shown that walking and running increase brain‐derived neurotrophic factor (BDNF) (Cho et al. 2012) and the effects of diet on the brain are integrated with exercise (Wu et al. 2008; Gomez‐Pinilla 2011; Hötting et al. 2016; Mandolesi et al. 2017; Kokubun, Nemoto et al. 2024). Consistent with these findings, the field of health science is beginning to reveal the relationship between diet and exercise and cognitive and social intelligence (Serra et al. 2020; González‐Valero 2020; Dominguez et al. 2021). Therefore, CQ, a social intelligence related to learning and brain function (Hedden et al. 2008; Chang 2017; Wang and Goh 2020), may have LS variables as predictors. CQ developers previously claimed that CQ is related to brain conditions (Rockstuhl et al. 2010; Ang et al. 2015). Recently, Kokubun, Nemoto, et al. (2025) developed a psychological model in which CQ predicts LS according to SRL theory and cross‐sectionally verified it. However, if we make a neuroscientific prediction based on the above arguments, the causal relationship is reversed, and it is possible that LS predicts CQ, as Kokubun, Nemoto, et al. (2023) showed in their previous simplified model consisting of diet and exercise.

CQ is related to outcomes such as cross‐cultural adaptation, performance, and turnover intentions (Jyoti and Kour 2017; Nunes et al. 2017; Chew et al. 2021; Ren et al. 2021; Setti et al. 2022). In addition, diet and sleep are related to the health and performance of expatriates and people of different origins (Truman et al. 2011; Fujimoto 2016). For example, a survey conducted by Truman et al. (2011) found that 36% of expatriates living abroad had sleep problems, compared to 24% of people living in the home country. Furthermore, it was shown that these problems worsen EP and interpersonal relationships (Truman et al. 2011). The issue of food is not easy either because adapting to local food and practicing a healthy diet are completely different in meaning. For example, previous research has shown that diabetes is much more common in Japanese Americans than in general Japanese or Americans (Fujimoto 2016). This means that adapting to the local LS may be harmful to one's health. At the same time, LS, including health and recreation, is related to the success or failure of expatriates' work and tasks (Naithani 2016; Prestes et al. 2016). For example, a study of 118 culturally diverse expatriates working in Europe showed that conflict between personal and work life was related to their health concerns (Grant‐Vallone and Ensher 2001). However, no research has shown the relationship between LS, CQ, and EP in an integrated manner. Given the neuroscientific relationship between LS and CQ, it is reasonable to consider that it is CQ, not LS, that is directly related to EP.

From the above discussion, LS can be considered as antecedents of CQ, and CQ can be considered as antecedents of EP, respectively. Therefore, CQ is thought to mediate the relationship between LS and EP. Furthermore, if such a relationship exists as a causal relationship rather than a correlational one, changes in CQ are thought to be accompanied by changes in LS and EP. In other words, changes in the mediator CQ resulting from an intervention should be accompanied by changes in LS as a cause and changes in EP as a consequence. This leads to the following two hypotheses that will be tested in this study.

Hypothesis 1 (H1): CQ mediates the relationship between LS and EP.

Hypothesis 2 (H2): Changes in CQ are accompanied by changes in LS and EP.

H1 will be tested through cross‐sectional analysis. H2 will be tested through longitudinal analysis to show that the relationship in H1 is robust. Below, we analyze the relationship between LS, CQ, and EP through two studies. Study 1 is a cross‐sectional analysis of participants recruited from among expatriates, and Study 2 is a longitudinal analysis of participants recruited from among the participants in Study 1 in the United States.

## 3 Materials and Method

Participants. In Study 1, a web survey was distributed and collected from October 24, 2023 to March 25, 2024 using the authors' personal networks to target expatriates stationed in Asia and the United States. Participants were required to be either (1) expatriates dispatched from the Japanese headquarters or (2) locally hired expatriates with at least one subordinate. The response time was approximately 15 min. Respondents were not paid a fee, but were given a simple report that allowed them to compare their results with other participants as an incentive to participate. A total of 184 people, 12 women and 172 men aged between 23 and 76, stationed in eight countries and regions in the east and west (China 125, Indonesia 2, Malaysia 9, Singapore 2, Taiwan 5, Thailand 9, United States 31, and Vietnam 1) participated, and the data from all of them were considered valid responses. One person's age could not be calculated because the date of birth was not filled in properly, so we used the average of the other participants instead.

In Study 2, volunteers were recruited from among the participants in Silicon Valley, USA in Study 1, and 21 Japanese expatriates aged 29–61 (17 men and 4 women) became the study subjects. After answering the online questionnaire (pre‐survey) administered in Study 1, participants freely used the smartphone app for 2 weeks, after which they answered the same questionnaire again (post‐survey). In this study, 15 people aged 32–56 (12 men and 3 women) were surveyed, excluding six who dropped out midway through.

**FIGURE 2:**
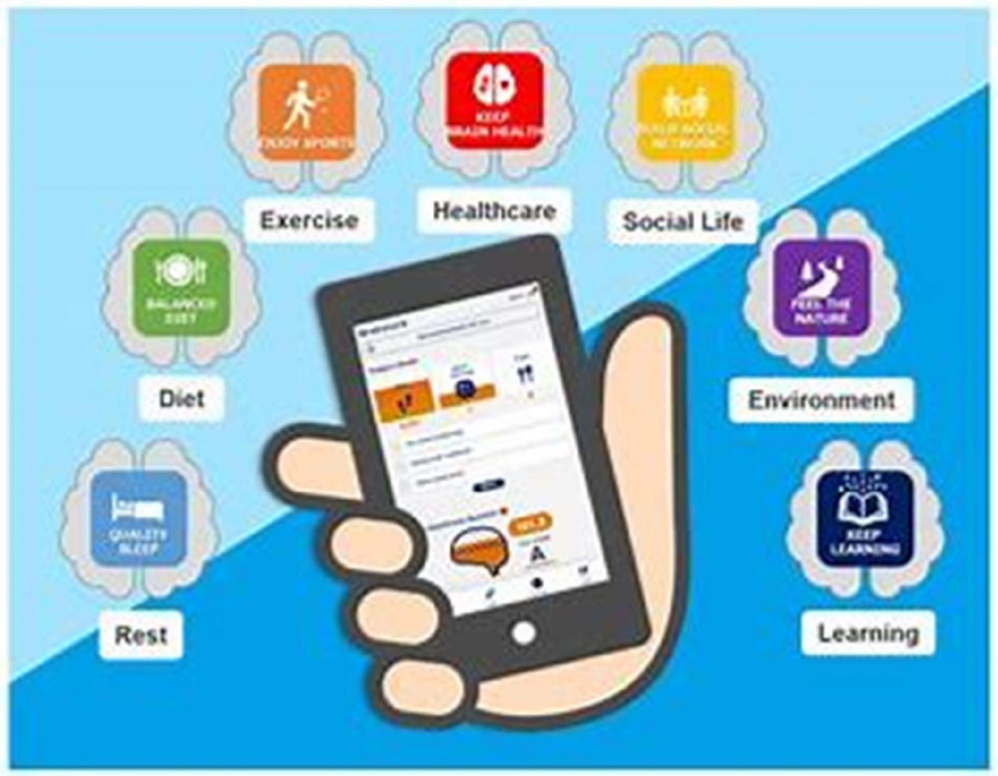
Conceptual diagram of Braincure retrieved from Businesswire (2023).

The intervention group trained “freely” every day using the beta version of “Braincure” developed by bspr Inc. (Brain Impact Association 2023) based on the BHQ Actions (Brain Impact Association 2024). In other words, participants were able to use the app as they pleased, without being restricted by time or method. Braincure allows users to know their estimated brain health by inputting their daily LS activities related to healthcare, social life, learning, exercise, environment, rest, and diet. Braincure also supports users in maintaining and improving their brain health by encouraging them to take actions such as exercising, improving their diet, and reducing stress (Businesswire 2023). For example, to calculate how much exercise a user is lacking, the app has the function of counting the number of steps the user takes, calculating dietary imbalances from the user's food records, and notifying the user of the value (Figure 2). In a previous brain science study, 35 healthy adult men and women used an app with some of the same functions of Braincure as they pleased for 1 month without time or method restrictions under similar conditions to the current study and a significant improvement was observed in fractional anisotropy (FA), an index reflecting the microstructure of the brain (Kokubun et al. 2024).

The reason for not restricting or monitoring the way and circumstances in which the app was used is to reduce the impact of a decrease in the enjoyment of using the app on its effectiveness. Recent studies have shown that the hassle of using the app and the fear of confidential information being obtained may reduce motivation to use health‐related apps (Kim and Lee 2022). In addition, the purpose of Study 2 is not to verify the performance of the smartphone app, but to confirm that when a certain health intervention triggers an improvement in CQ, CQ plays the role of a mediator, that is, it is accompanied by improvements in LS and EP. Therefore, no guidelines were set for the subject group or the use of the smartphone app, and no monitoring was conducted.

This study was approved by the Ethics Committee of Institute of Science Tokyo (Approval Number 2023137) and was conducted following the institutes' guidelines and regulations. All participants provided written informed consent before participation, and their anonymity was maintained.

## 4 Psychological Measures

### 4.1 Cultural Intelligence

The CQ scale was adopted from Ang et al. (2007). A total of 20 CQ items comprised four facets: Metacognitive CQ (four items, e.g., “I check the accuracy of my cultural knowledge as I interact with people from different cultures”); Cognitive CQ (six items, e.g., “I know the rules for expressing non‐verbal behavior in other cultures”); Motivational CQ (five items, e.g., “I enjoy interacting with people from different cultures”); and Behavioral CQ (five items, e.g., “I change my verbal behavior when a cross‐cultural interaction requires it”). All items were rated on a 7‐point Likert scale (1 = *strongly disagree*; 7 = *strongly agree*), and the items were averaged to calculate the value of each variable. In addition, in Study 2, which did not include latent variables, the average of the 20 items was calculated and used as the Overall CQ (20 items, *α*  = 0.898).

### 4.2 Lifestyle

As a variable representing LS, we used the BHQ actions scale, which consists of seven subsets: “Healthcare,” “Social life,” “Learning,” “Exercise,” “Environment,” “Rest,” and “Diet” (Kokubun, Toyama, et al. 2023; Kokubun, Nemoto, et al. 2025). For details on the development procedures for each scale and the questionnaire, please refer to Kokubun, Nemoto, et al. (2025).

Healthcare: After the introductory sentence, “To what extent are you interested in your own health? Please tell us whether or not the following applies to the actions you are taking,” we presented four questions: “1. I am interested in health,” “2. I collect information about health,” “3. I regularly check my health status,” and “4. I am taking steps to improve my health,” and asked participants to choose from two options for each: “1. Applies” or “2. Does not apply.” After that, we assigned a score of 1 if the number of “1. Applies” was 0, 2 if 1, 3 if 2, 4 if 3, and 5 if 4.

Social life: After the introductory statement “How many social relationships do you have? Please tell us whether or not any of the following apply to you,” five questions were presented: “1. Living with spouse,” “2. Living with family members other than spouse,” “3. Interacting with friends,” “4. Participating in local group activities,” and “5. Working.” For each question, participants were asked to choose from two options: “1. Applies” or “2. Does not apply.” After that, participants were assigned a score of 1 if they answered “1. Applies” to 0 or 1 question, 2 if they answered “2,” 3 if they answered “3,” 4 if they answered “4,” and 5 if they answered “5.”

Learning: The question “How often have you engaged in hobbies or learning in the past year (how much time do you spend on hobbies or learning on weekdays)?” was presented, and participants were asked to choose from five options: “1: less than once a year,” “2: several times a year,” “3: several times a month,” “4: several times a week,” and “5: daily/almost daily.” They were then assigned a score from 1 to 5.

Exercise: The question “How many times a week do you exercise for 30 minutes or more?” was presented, and participants were asked to choose from five options: “1: Never,” “2: Once a week,” “3: Twice a week,” “4: Three times a week,” and “5: Every day/almost every day.” They were then assigned a score from 1 to 5.

Environment: The question “How much time do you have per week to go outside and experience nature?” was presented, and participants were asked to choose from five options: “1: Not at all,” “2: Less than 1 hour per week,” “3: 1–2 hours per week,” “4: 2–3 hours per week,” and “5: 3 hours or more per week.” They were then assigned a score from 1 to 5.

Rest: The question “Please tell us whether this applies to your sleep during the last week” was presented, and participants were asked to choose from five options: “1. I fall asleep easily,” “2. I sleep soundly until morning,” “3. I don't take naps or they are short (less than 30 minutes),” “4. I sleep at the same time,” and “5. I sleep 7 to 8 hours a night.” They were then assigned a score of 1 to 5.

Diet: The question “Please select the number of items consumed in meals last week” was presented, and participants were asked to choose between “1. Applies” and “2. Does not apply” for each of 14 items, including “I ate green leafy vegetables 6 or more times a week.” After that, participants were assigned a score of 1 if 3 or fewer items applied, a score of 2 if 4–6 items applied, a score of 3 if 7–8 items applied, a score of 4 if 9–12 items applied, and a score of 5 if 13 or more items applied.

### 4.3 Expatriate Performance

Cultural adjustment. Adopted from Black and Stephens (1989). A total of 14 items make up the following three facets: General adjustment (seven items, e.g. “Living conditions in general”); interaction adjustment (four items, e.g. “Socializing with host nationals”); and work adjustment (three items, e.g. “Specific job responsibilities”). All items are rated on a 7‐point Likert scale (1 = not adjusted at all; 7 = very well adjusted), and the value of each variable is calculated by the average of the items.

Performance. Adopted from Caligiuri (1997). A total of 12 items make up the following four facets: Overall performance (two items, e.g. “your performance of your job responsibilities as an expatriate”); Technical performance (one item, “your technical performance on this expatriate assignment”); Contextual/Managerial performance (five items, e.g. “your ability to foster organizational commitment in the foreign subsidiary”); Expatriate‐Specific performance (four items, e.g. “your effectiveness at training your expatriate or host national replacement”). All items were rated on a 5‐point Likert scale (1 = *unsatisfactory* or *poor*; 7 = *exceptional* or *outstanding*), and the value of each variable was calculated by the average of the items.

Intentions to complete the assignment. Adopted from Kraimer and Wayne (2004). A total of three items constitutes one variable (e.g., “I intend to stay for the entire expected length of my expatriate assignment”). All items were rated on a 7‐point Likert scale (1 = *strongly disagree*; 7 = *strongly agree*), and the value of each variable was calculated as the average of the items.

For the CQ, the Japanese version by Nakao (2019) was used. However, for the three scales of intercultural adaptation, performance, and intentions to complete the assignment, Japanese versions were not available at the time of this study. Therefore, the authors and one international student from an English‐speaking country translated them into Japanese using the back‐translation method.

### 4.4 Item Parceling

Previous studies have shown that expatriates' cross‐cultural adaptation is related to performance (L. Y. Lee and Sukoco 2010; Malek and Budhwar 2013) and that cross‐cultural adaptation is related to premature termination of expatriates' assignments (Caligiuri 1997, Kraimer and Wayne 2004). There is also a wealth of research showing the relationship between these outcomes and CQ (Jyoti and Kour 2017; Nunes et al. 2017; Chew et al. 2021; Setti et al. 2022). Therefore, in this study, we thought that it would be easier to interpret the model by using a second‐order construct that combines these outcomes, rather than unnecessarily complicating the model by treating them individually. In the following analysis, LS, CQ, and EP are treated as latent variables, and the subsets that make up these, seven, four, and eight, respectively, are used as manifest variables. In other words, we treat LS, CQ, and EP as parceled items with a subset of each as the first‐order construct (Hau and Marsh 2004).

According to Rhemtulla (2016), if the focus of the study is to verify the measurement model, that is, to verify how accurately the questionnaire measures the constructs that you want to know about, parceling is not desirable. On the other hand, if the study is interested in verifying the relationships between constructs, parceling is likely to lead to appropriate inferences by simplifying the structural equations (Rhemtulla 2016). Since Study 1 uses already established psychological scales to verify the relationships between constructs, it corresponds to the latter case, and parceling is considered appropriate.

### 4.5 Control Variables

In Study 1, area (United States = 1; Asian countries = 0), age (years old), educational background (years), overseas work experience (months), and sex (male = 1; female = 0) were used as control variables.

## 5 Data Analysis

In Study 1, a mediation test was conducted to show that CQ significantly mediated the relationship between LS and EP. To do so, SEM was conducted with LS, CQ, and EP as latent variables of a second‐order construct. Next, in Study 2, to confirm the results of Study 1, a paired *t*‐test was used to measure the change in each variable before and after the intervention. In addition, a correlation analysis was conducted using the range of change in the variables to confirm that CQ played a role as a mediator. All statistical analyses have been performed using IBM SPSS Statistics/AMOS Version 26 (IBM Corp., Armonk, NY, USA).

## 6 Results

Before proceeding with the main analysis, we used Harman's single factor analysis to check whether the variance in the data could be mainly attributed to a single factor, and also used confirmatory factor analysis (CFA) to test whether the factors were related to the measurement. First, Harman's factor analysis showed that only 27.6% of the variance could be explained by a single factor, which was < 50%. Thus, it was established that the data were not affected by common method variance (Podsakoff et al. 2003). Next, we conducted a CFA to evaluate the fit of the model. Here, we followed Kline (2023) who advocates the use of chi‐square test, comparative fit index (CFI), root mean square error of approximation (RMSEA), and standardized root mean square residual (SRMR). The acceptable range of index values is as follows: chi‐square value from 0 to 3 (McIver and Carmines 1981); CFI above 0.90 (Hu and Bentler 1999); RMSEA < 0.08 (Schermelleh‐Engel et al. 2003); SRMR < 0.08 (Hu and Bentler 1999). As Table 1 shows, all multidimensional structures meet the acceptable requirements. In addition, for the psychometric scale CQ, we tested the average variance extracted (AVE) and composite reliability (CR). AVE: Metacognitive CQ = 0.619; Cognitive CQ = 0.579; Motivational CQ = 0.634; Behavioral CQ = 0.550. CR: Metacognitive CQ = 0.865; Cognitive CQ = 0.892; Motivational CQ = 0.896; Behavioral CQ = 0.856. All subsets exceeded the AVE standard value of 0.50 (Fornell and Larcker 1981) and the CR standard value of 0.70 (Nunnally and Bernstein 1994), confirming high convergent validity and internal consistency, respectively.

**TABLE 1:** Summary of confirmatory factor analysis.

Construct	*X* ^2^	df	*X* ^2^/df	*p*	CFI	RMSEA	SRMR
LS	10.538	10	1.054	0.395	0.995	0.017	0.048
CQ	2.400	2	1.200	0.301	0.997	0.033	0.024
EP	19.408	15	1.294	0.196	0.993	0.040	0.034

*Note*: A second‐order construct.

Abbreviations: CFI = comparative fit index; CQ = cultural intelligence; df = degree of freedom; LS = lifestyle; EP = expatriate performance; RMSEA = root mean square error of approximation; SD = standard deviation; SRMR = standardized root mean square residual.

Table 2 shows descriptive statistics. The first four columns show the mean and SD for Studies 1 and 2. An independent *t*‐test was conducted to confirm the difference in distribution between the two. There were significant differences in age, education, length of work abroad, as well as social life and metacognitive CQ. The fifth column and onwards show correlation coefficients. The bottom left shows the results for Study 1, and the top right shows the results for Study 2. The Cronbach's alpha value for Study 1 is shown in parentheses on the diagonal. All the alpha values ​​exceeded the standard value of 0.70 (Nunnally and Bernstein 1994), confirming high reliability.

**TABLE 2:** Descriptive statistics.

		Study 1		Study 2		Correlation coefficient																						
		Mean	SD	Mean	SD	1	2	3	4	5	6	7	8	9	10	11	12	13	14	15	16	17	18	19	20	21	22	23
1	LS1: Healthcare	4.140	1.132	4.670	0.488		−0.250	0.303	0.433	0.078	0.099	0.088	0.125	0.217	0.105	0.273	0.310	0.700^**^	0.533^*^	0.249	0.322	0.220	0.319	−0.362	0.118	0.101	0.281	0.000
2	LS2: Social life	3.010	0.978	3.670^*^	0.976	0.143		−0.434	0.361	0.509	−0.025	0.373	−0.098	−0.236	−0.210	−0.701^**^	−0.334	−0.340	−0.436	−0.539^*^	−0.253	−0.220	−0.271	0.115	0.143	−0.151	0.162	0.530^*^
3	LS3: Learning	3.460	0.957	3.470	1.125	0.197^**^	0.044		0.213	0.309	0.068	−0.076	0.470	0.391	0.401	0.291	0.740^**^	0.435	0.174	−0.115	−0.124	−0.264	−0.136	−0.242	−0.164	0.227	0.146	−0.245
4	LS4: Exercise	2.780	1.177	2.800	1.014	0.278^***^	0.006	0.259^***^		0.644^**^	0.171	0.127	0.077	−0.068	−0.162	−0.090	0.011	0.244	−0.028	−0.287	−0.027	−0.134	−0.028	0.142	0.223	−0.039	0.353	0.408
5	LS5: Environment	3.340	1.337	3.130	1.246	0.084	0.057	0.270^***^	0.429^***^		0.042	0.120	0.327	0.199	0.115	−0.165	0.225	0.189	0.067	−0.201	−0.040	−0.066	−0.054	0.200	−0.069	−0.189	0.209	0.332
6	LS6: Rest	3.230	1.238	3.130	0.990	0.125	0.270^***^	−0.030	−0.045	0.023		−0.043	0.096	0.406	0.283	0.369	−0.107	−0.002	−0.053	0.155	0.358	0.152	0.168	−0.356	0.294	−0.288	0.541^*^	0.244
7	LS7: Diet	3.060	0.987	3.330	1.113	0.261^***^	0.226^**^	0.063	0.190^**^	0.109	0.042		−0.078	0.147	0.092	−0.376	−0.228	0.174	−0.016	−0.364	−0.020	0.113	0.280	−0.512	−0.364	0.044	0.366	−0.155
8	CQ1: Metacognitive CQ	5.088	1.016	5.667^*^	0.686	0.187^*^	0.009	0.069	0.096	0.056	0.008	0.148^*^	(0.809)	0.438	0.343	0.229	0.490	0.242	0.298	0.015	−0.090	0.052	−0.011	−0.126	−0.072	−0.125	0.206	−0.126
9	CQ2: Cognitive CQ	4.577	1.196	4.433	1.239	0.161^*^	0.142	0.179^*^	0.109	0.220^**^	0.083	0.312^***^	0.233^**^	(0.898)	0.731^**^	0.566^*^	0.449	0.442	0.470	0.374	0.455	0.482	0.539^*^	−0.288	0.042	0.122	0.243	−0.005
10	CQ3: Motivational CQ	5.587	0.949	5.560	0.836	0.173^*^	0.194^**^	0.241^**^	0.057	0.126	0.109	0.289^***^	0.246^**^	0.539^***^	(0.897)	0.338	0.549^*^	0.575^*^	0.421	0.250	0.381	0.435	0.390	−0.092	0.191	0.449	0.387	0.099
11	CQ4: Behavioral CQ	4.970	1.019	5.080	0.751	0.211^**^	0.144	0.088	0.044	0.106	0.055	0.280^***^	0.269^***^	0.488^***^	0.391^***^	(0.845)	0.384	0.481	0.521^*^	0.694^**^	0.570^*^	0.483	0.537^*^	0.019	−0.034	0.021	−0.037	−0.312
12	EP1: General adjustment	5.477	0.823	5.467	0.629	0.196^**^	0.208^**^	0.219^**^	0.112	0.160^*^	0.177^*^	0.137	0.181^*^	0.373^***^	0.477^***^	0.209^**^	(0.862)	0.588^*^	0.245	0.071	0.014	−0.100	−0.087	−0.074	−0.098	0.313	−0.127	−0.192
13	EP2: Interaction adjustment	5.081	1.044	4.613	0.934	0.228^**^	0.180^*^	0.113	0.184^*^	0.255^***^	0.118	0.270^***^	0.192^**^	0.500^***^	0.614^***^	0.369^***^	0.504^***^	(0.883)	0.691^**^	0.354	0.464	0.471	0.545^*^	−0.131	−0.098	0.353	0.277	−0.214
14	EP3: Work adjustment	5.511	0.961	5.440	1.208	0.189^*^	0.118	0.124	0.084	0.092	0.128	0.298^***^	0.195^**^	0.396^***^	0.500^***^	0.369^***^	0.429^***^	0.571^***^	(0.913)	0.776^**^	0.735^**^	0.815^***^	0.803^***^	0.068	−0.132	0.059	0.333	−0.111
15	EP4: Overall performance	3.777	0.687	3.800	0.882	0.195^**^	0.095	0.099	0.102	0.023	0.076	0.149^*^	0.291^***^	0.316^***^	0.356^***^	0.327^***^	0.318^***^	0.365^***^	0.604^***^	(0.902)	0.848^***^	0.810^***^	0.723^**^	0.164	−0.096	−0.212	0.071	−0.117
16	EP5: Technical performance	3.790	0.749	3.870	1.060	0.094	0.069	−0.007	0.102	−0.021	0.047	0.224^**^	0.215^**^	0.256^***^	0.316^***^	0.355^***^	0.217^**^	0.341^***^	0.506^***^	0.651^***^		0.872^***^	0.856^***^	0.061	−0.063	−0.148	0.451	0.098
17	EP6: Contextual/Managerial performance	3.745	0.656	3.760	0.797	0.165^*^	0.123	0.083	0.167^*^	0.086	0.028	0.221^**^	0.265^***^	0.433^***^	0.471^***^	0.373^***^	0.289^***^	0.495^***^	0.658^***^	0.718^***^	0.556^***^	(0.862)	0.942^***^	0.133	−0.040	−0.035	0.369	0.017
18	EP7: Expatriate‐ Specific performance	3.536	0.635	3.587	0.612	0.268^***^	0.177^*^	0.139	0.179^*^	0.141	0.055	0.257^***^	0.256^***^	0.522^***^	0.478^***^	0.368^***^	0.363^***^	0.547^***^	0.565^***^	0.597^***^	0.442^***^	0.734^***^	(0.784)	0.010	−0.128	0.039	0.392	−0.068
19	EP8: Intentions to complete the assignment	5.796	1.276	6.111	0.989	−0.055	0.086	0.180^*^	0.040	0.022	−0.025	−0.063	−0.039	−0.050	0.182^*^	0.075	0.166^*^	0.092	0.115	0.189^*^	0.071	0.150^*^	0.085	(0.747)	0.290	0.215	−0.235	0.407
20	Age	49.495	9.982	43.730^*^	7.005	0.110	−0.132	−0.134	0.240^**^	0.217^**^	0.046	0.217^**^	0.243^**^	0.165^*^	0.002	0.146^*^	−0.034	0.176^*^	0.215^**^	0.178^*^	0.194^**^	0.243^**^	0.156^*^	−0.095		0.460	0.122	0.744^**^
21	Education	15.890	1.934	17.400^**^	1.454	0.347^***^	0.156^*^	0.156^*^	0.042	−0.034	0.018	0.078	0.162^*^	0.148^*^	0.233^**^	0.068	0.168^*^	0.161^*^	0.150^*^	0.160^*^	0.077	0.215^**^	0.322^***^	0.066	−0.092		−0.056	0.142
22	Length of work abroad	106.830	92.155	53.270^***^	40.821	0.035	−0.001	−0.080	0.251^**^	0.090	−0.109	0.202^**^	0.180^*^	0.346^***^	0.059	0.129	0.112	0.227^**^	0.234^**^	0.160^*^	0.217^**^	0.351^***^	0.317^***^	−0.061	0.481^***^	0.036		0.316
23	Sex	0.930	0.248	0.800	0.414	−0.123	−0.111	−0.034	0.045	0.100	0.013	−0.029	0.007	−0.103	−0.101	−0.095	−0.08	−0.075	−0.012	0.027	0.072	−0.036	−0.055	−0.112	0.274^***^	−0.073	0.030	
24	Area	0.170	0.375	1.000^***^	0.000	0.098	0.191^**^	0.071	−0.065	−0.048	−0.001	−0.042	−0.014	−0.122	−0.009	−0.047	−0.023	−0.190^**^	−0.093	0.030	−0.028	−0.091	0.041	0.157^*^	−0.297^***^	0.350^**^	−0.273^***^	0.175^*^

*Note*: *n*  =  184.

Abbreviations: CQ = cultural intelligence; EP = expatriate performance; LS = lifestyle; SD = standard deviation.

^*^ *p* < 0.05; ^**^ *p* < 0.01; ^***^ *p* < 0.001.

The correlation coefficients are shown in the bottom left for Study 1 and in the top right for Study 2. The numbers in parentheses on the diagonal are Cronbach's alphas for Study 1.

**FIGURE 3:**
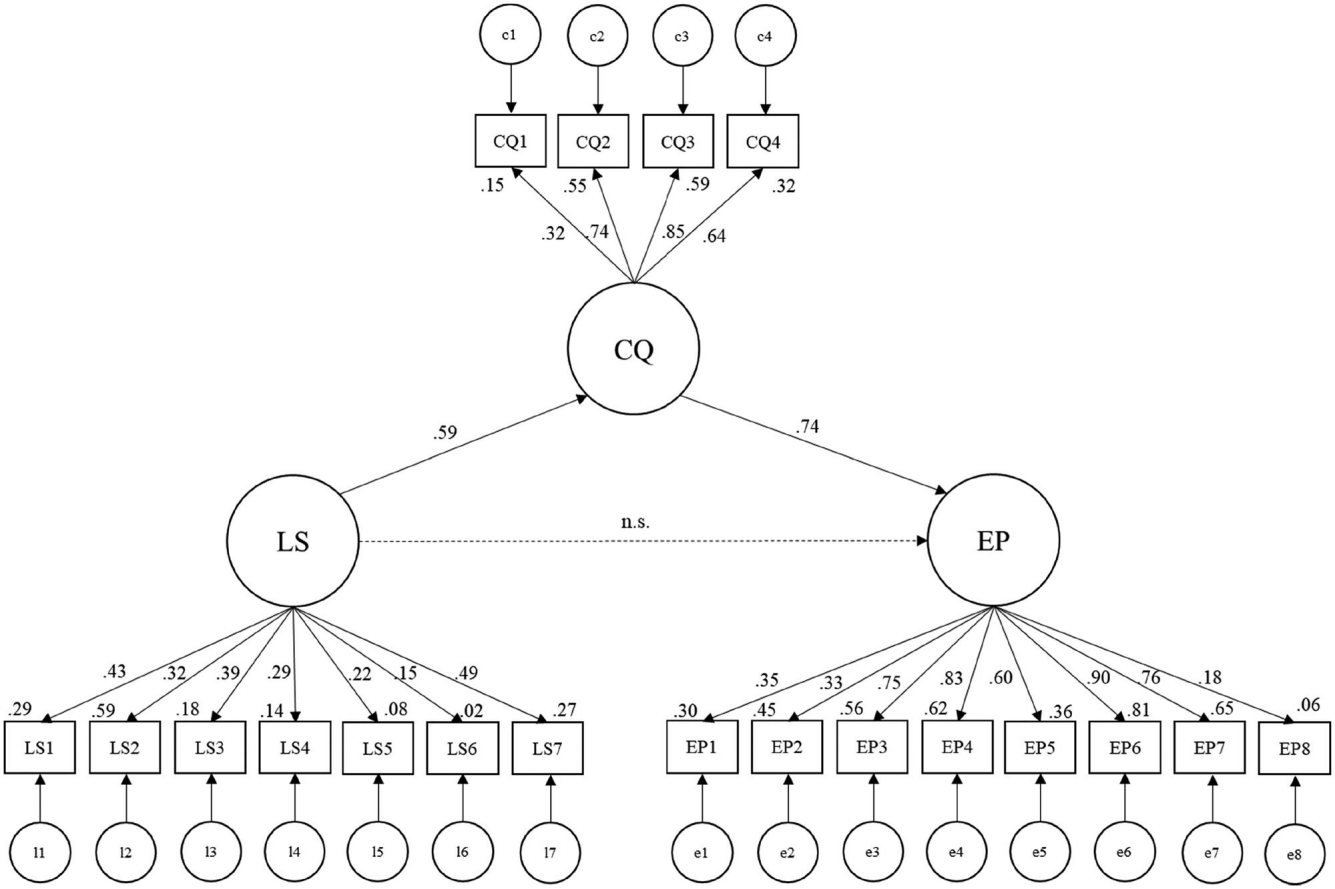
Results of covariance structure analysis *n*  =  184; All the solid paths were significant at *p* < 0.05. The dashed paths were insignificant (*p* > 0.05). The figures are standardized (*β*) and controlled for area, age, education, and overseas experience. Modification indices were used to improve the fit. Sex was omitted from the final model because it was not related to any of the variables. Correlations between variables are omitted in the figure (mainly correlations between error terms of manifest variables with a common latent variable. Available upon request). CQ = cultural intelligence; EP = expatriate performance, LS = lifestyle; n.s. = not significant.

As shown in Figure 3, we conducted a mediation test using 5000 bootstrap samples, maximum likelihood estimators, and 95% bias‐corrected confidence intervals. Overall, the structural mediation model explained the data well (*X* ^2^ = 223.484, df = 199, *X* ^2^/df = 1.123, *p* = 0.112, CFI = 0.981, RMSEA = 0.026, SRMR = 0.055). Next, we evaluated the mediating role of CQ on the relationship between LS and EP. The results showed that the indirect effect of LS on EP was positive and significant (*b* = 0.433, *p* = 0.000), supporting H1. On the other hand, the direct effect of LS on EP was positive and significant before CQ was entered as a mediator (*b* = 0.832, *p* = 0.023), but was not significant in the presence of the mediator (*b* = −0.117, *p* = 0.611). Thus, CQ was shown to fully mediate the relationship between LS and EP. A summary of the mediation analysis is shown in Table 3.

**TABLE 3:** Summary of mediation analysis.

Relationship	Direct	*p* value	Indirect	95% CI		*p* value	Conclusion
	Effect		Effect	Upper	Lower		
LS → CQ → EP	−0.117	0.611	0.433	0.203	0.680	0.000	Full mediation

*Note*: Numbers are unstandardized values (*b*).

Table 4 shows the results of Study 2, that is, the values ​​of various indices after the intervention. Participants' sleep, cognitive CQ, behavioral CQ, overall CQ, and intentions to complete the assignment showed significant improvements at the 5% level. Table 5 shows the correlation coefficients between the change before and after the intervention. ΔCognitive CQ was correlated with ΔPerformance (*r* = 0.600, *p* = 0.018), ΔBehavioral CQ was correlated with ΔTechnical performance (*r* = 0.691, *p* = 0.004), ΔOverall CQ was correlated with ΔLearning (*r* = 0.670, *p* = 0.006), ΔPerformance (*r* = 0.682, *p* = 0.005), ΔExchange adaptation (*r* = 0.562, *p* = 0.029), ΔContextual‐managerial performance (*r* = 0.527, *p* = 0.044), and ΔExpatriate‐specific performance (*r* = 0.584, *p* = 0.022), supporting H2.

**TABLE 4:** Values in indicators after the intervention.

	Post intervention				
	Mean	SD	*t*	*p*	Cohen's *d*
LS					
LS1: Healthcare	4.467	0.834	1.146	0.271	0.301
LS2: Social life	3.667	1.047	0.000	1.000	0.006
LS3: Learning	3.400	0.986	0.435	0.670	0.118
LS4: Exercise	2.933	1.100	0.807	0.433	0.208
LS5: Environment	3.000	1.309	0.619	0.546	0.156
LS6: Rest	3.667^*^	0.817	4.000	0.001	1.040
LS7: Diet	3.200	1.146	0.354	0.728	0.089
*CQ*					
CQ1: Metacognitive CQ	5.820	0.610	0.735	0.474	0.190
CQ2: Cognitive CQ	4.953^**^	1.076	2.435	0.029	0.628
CQ3: Motivational CQ	5.613	0.754	0.254	0.803	0.066
CQ4: Behavioral CQ	5.480^**^	0.692	2.523	0.024	0.652
Overall CQ	5.447^**^	0.651	2.293	0.038	0.592
*EP*					
EP1: General adjustment	5.407	0.767	0.388	0.704	0.100
EP2: Interaction adjustment	5.120	0.829	2.063	0.058	0.533
EP3: Work adjustment	5.600	0.723	0.615	0.549	0.159
EP4: Overall performance	4.000	0.681	1.146	0.271	0.296
EP5: Technical performance	4.067	0.704	1.000	0.334	0.254
EP6: Contextual/Managerial performance	3.827	0.477	0.393	0.700	0.101
EP7: Expatriate‐ Specific performance	3.853	0.297	1.865	0.083	0.481
EP8: Intentions to complete the assignment	6.473^**^	0.681	2.387	0.032	0.616

*Note*: *n* = 15.

Abbreviations: CQ = cultural intelligence; EP = expatriate performance; LS = lifestyle.

^*^ *p* < 0.01

^**^ *p* < 0.05.

**TABLE 5:** Correlation of change.

	CQ (Mediator)				
	ΔMetacognitive CQ	ΔCognitive CQ	ΔMotivational CQ	ΔBehavioral CQ	ΔOverall CQ
LS					
ΔHealthcare	−0.048	0.415	0.229	0.241	0.304
ΔSocial life	0.241	0.298	−0.066	0.218	0.264
ΔLearning	0.497	0.426	0.719^*^	0.313	0.670^*^
ΔExercise	0.271	0.600^**^	0.508	0.509	0.682^*^
ΔEnvironment	0.235	0.476	0.285	0.195	0.446
ΔRest	−0.186	0.178	0.302	0.315	0.199
ΔDiet	0.069	0.232	0.055	0.208	0.167
EP					
ΔGeneral adjustment	0.500	0.128	0.255	0.258	0.379
ΔInteraction adjustment	0.345	0.461	0.538^**^	0.259	0.562^**^
ΔWork adjustment	0.346	0.257	0.398	0.371	0.452
ΔOverall performance	−0.180	−0.154	0.291	0.482	0.095
ΔTechnical performance	0.020	0.212	0.481	0.691^*^	0.464
ΔContextual/Managerial performance	0.342	0.376	0.437	0.361	0.527^**^
ΔExpatriate‐Specific performance	0.291	0.422	0.474	0.512	0.584^**^
ΔIntentions to complete the assignment	−0.082	−0.119	−0.194	0.103	−0.096

*Note*: *n* = 15.

Abbreviations: CQ = cultural intelligence; EP = expatriate performance; LS = lifestyle.

^*^ *p* < 0.01

^**^ *p* < 0.05.

## 7 Discussion

In this paper, Study 1 showed that CQ of 184 expatriates mediated LS and EP, and Study 2 showed that improvements in CQ of 15 of these expatriates were accompanied by improvements in LS and EP. These results are partially consistent with previous research showing that LS is correlated with CQ (Kokubun et al. 2024; Kokubun, Nemoto, et al. 2025) but for the first time showed that CQ mediates the relationship between LS and EP. The consistency of the results of Study 1 and Study 2 suggests that the robustness of this study is at a certain level. However, Study 2 was an exploratory pilot study and its small sample size and lack of a control group suggest low reproducibility and the possibility of variation due to other factors, and these should be discounted in evaluation.

Many studies have shown that CQ is an essential skill for expatriates of multinational companies and people who deal with different cultures, and that CQ is therefore effective in intercultural adaptation and maintaining and improving performance (Jyoti and Kour 2017; Nunes et al. 2017; Chew et al. 2021; Setti et al. 2022). Therefore, in addition to special training to improve CQ (Earley and Peterson 2004; Rehg et al. 2012; Philip et al. 2023), the relationship between LS and CQ has been elucidated from the perspectives of neuroscience and psychology (Kokubun, Nemoto, et al. 2023; Kokubun, Nemoto, et al. 2025). Furthermore, because previous studies have shown a relationship between LS and EP (Truman et al. 2011; Fujimoto 2016; Naithani 2016; Prestes et al. 2016), it was expected that CQ would mediate the relationship between LS and EP, which motivated this study. Therefore, this study, which showed that improving diverse LS improves CQ and indirectly improves EP among expatriates working in a cross‐cultural environment, adds value to traditional health research and cross‐cultural research.

Among them, rest, which showed a direct effect of the intervention, is one of the factors that affect individual performance, because subjective sleep quality has been shown to be related to self‐reported reward expectancy (Markarian et al. 2013; Palmer and Alfano 2017). Previous studies have shown that after a night of sleeplessness, individuals find it harder to delay gratification (Killgore et al. 2008) and are therefore more likely to choose smaller rewards that require less effort over larger rewards that require more effort (Libedinsky et al. 2013). As a result, insomniacs have been shown to be more likely than good sleepers to take time off work, cancel appointments or meetings, or skip social activities due to fatigue or sleepiness without considering the consequences (Harvey 2002; Hood et al. 2011; Deng et al. 2022). Therefore, the results of this study, which showed that increases in sleep is accompanied by improvements in CQ, the social intelligence needed to understand different cultures and put that into action, and increases in the will to complete tasks to continue working persevering, are reasonable.

In addition, increases in exercise, hobbies, and learning were correlated with increases in CQ. A systematic review has confirmed that exercise has a positive effect on self‐control, prosocial behavior, and interpersonal communication, and that exercise is more effective when done in a team than when done individually (Teixeira et al. 2012). Therefore, it can be inferred that some participants were more likely to be in a state where intercultural understanding was enhanced by improving their interpersonal interactions. A recent systematic review has confirmed that hobbies and learning can have multifaceted effects, such as improving happiness and maintaining health, by practicing them (Fancourt et al. 2021). There are also studies that show that certain hobbies and learning, such as reading, enhance empathy for others and cognitive ability (Kidd and Castano 2013). This is because when readers begin to empathize with a character, they begin to think about the character's goals and desires, rather than their own (Oatley 2016). Thus, with training, we can intentionally change our attention to the emotions of others (Weisz and Zaki 2018). Motivational CQ, which correlated with an increase in learning, refers to empathy for and a willingness to engage with different cultures (Ang et al. 2007). It is not surprising, therefore, that expatriates with such hobbies are more likely to have an empathy for and a strong desire to learn about the local culture.

In addition, diet, which was found to be correlated with CQ in Study 1, is consistent with a systematic review showing that a healthy diet reduces mental health problems such as depression, anxiety, and stress in college students (Solomou et al. 2023), and research showing that a balanced diet is associated with cognition and episodic memory (Guasch‐Ferre and Willett 2021). Episodic memory is the memory of an event, including the time, place, and emotions felt at the time. Therefore, people with unhealthy diets may be more likely to have vague memories of their intercultural experiences and the emotions they felt at the time, which may lead to a decrease in their knowledge and interest in intercultural experiences, and they may feel uncomfortable behaving appropriately during interactions.

Previous studies on the relationship between LS and EP were mainly conducted in domestic settings. On the other hand, studies on the relationship between CQ and EP were mainly conducted in international settings. Therefore, at the time of writing this study, the only study that clarified the relationship between LS and CQ was Kokubun, Nemoto, et al. 2023; Kokubun, Nemoto, et al. 2025). The current study was the first to show that CQ mediates the relationship between various LS and EP by combining cross‐sectional analysis and pilot longitudinal analysis. Therefore, this study is positioned in both streams of health science and cross‐cultural research, and at the same time, it makes a unique contribution to academia by bridging the two.

The results of this study may prompt a change in intervention methods to enhance CQ. Previous studies have emphasized cross‐cultural experiences, related training, and virtual technology as means to enhance CQ, but it has been pointed out that such methods often do not produce sufficient effects (Ang et al. 2007; Ott and Michailova 2018). Given that CQ is intelligence and is related to the brain, it is unreasonable not to consider LSs related to the brain. Managers in a position to manage expatriates may be able to solve various management problems in cross‐cultural environments by paying more attention to the health of their expatriates than ever before.

This idea can be connected to recent research attempts to position CQ within COR theory (Fu and Charoensukmongkol 2023; A. S. Y. Chen et al. 2024). In other words, positioning CQ as a psychological resource makes it possible to think not only about using CQ to compensate for the EP deficiencies of expatriates, but also about compensating for CQ deficiencies by recharging through LS. This makes it possible to treat the relationship between health and work in a cross‐cultural environment as a more intervenable subject in the context of health science.

## 8 Limitation

Like many other studies, this study has some problems to overcome. First, although the sample size of Study 1 is relatively large at 184, it was only for Japanese people and should be applied with caution to other countries. In addition, since it is a cross‐sectional study, it does not show the causal relationship between variables. Second, the sample size of the longitudinal study in Step 2 is only 15, which is insufficient in terms of generalizability. In addition, since there was no control group, guidelines for the use of the smartphone app were not set, and the use process was not monitored, it is difficult to evaluate the effect of the intervention or to propose specific intervention methods (however, as mentioned in the text, Step 2 was exploratory/pilot study and the purpose was not to verify the effect of the smartphone app, but to confirm the robustness of the results shown by the cross‐sectional analysis in Step 1 that improvements in CQ are accompanied by improvements in LS and EP). Therefore, future studies can verify and develop the results of this study by conducting intervention studies based on more rigorous methods with a larger sample size including business people of various nationalities, setting up a control group, guidelines, and monitoring.

## 9 Summary

Interest in CQ is growing as people's global activities increase. However, no research has been conducted to date that examines the relationship between CQ, LS, and EP in an integrated manner. The results of a cross‐sectional analysis using survey data from expatriates and a pilot longitudinal analysis showed that CQ mediates the relationship between LS and EP. This is the first study to demonstrate the possibility of using health science interventions to enhance CQ and increase people's international achievements.

## Author Contributions

**Keisuke Kokubun**: conceptualization, software, formal analysis, funding acquisition, writing–original draft, methodology, writing–review and editing. **Kiyotaka Nemoto**: writing–review and editing, methodology, data curation, resources. **Yoshinori Yamakawa**: conceptualization, funding acquisition, investigation, project administration, supervision.

## Ethics Statement

This study was approved by the Ethics Committee of Institute of Science Tokyo (Approval Number 2023137) and was conducted following the institute's guidelines and regulations. All participants provided written informed consent before participation, and their anonymity was maintained.

## Consent

All participants gave consent for the publication of the results of this study.

## Conflicts of Interest

The authors declare no conflicts of interest.

### Peer Review

The peer review history for this article is available at https://publons.com/publon/10.1002/brb3.70576

## Data Availability

The data that support the findings of this study are available on request from the corresponding author. The data are not publicly available due to privacy or ethical restrictions.
